# 恶性胸腔积液局部治疗研究进展

**DOI:** 10.3779/j.issn.1009-3419.2025.102.32

**Published:** 2025-08-20

**Authors:** Yanjun DU, Ping ZHAN, Tangfeng LV, Yong SONG

**Affiliations:** ^1^210002 南京，南京医科大学金陵临床医学院呼吸与危重症学科（杜彦君，展平，吕镗烽，宋勇）; ^1^Department of Respiratory and Critical Care Medicine, Jinling Clinical Medical College, Nanjing Medical University, Nanjing 210002, China; ^2^210002 南京，南京大学医学院附属金陵医院呼吸与危重症学科（展平，吕镗烽，宋勇）; ^2^Department of Respiratory and Critical Care Medicine, Nanjing Jinling Hospital, Affiliated Hospital of Medical School, Nanjing University, Nanjing 210002, China; ^3^210002 南京，南京中医药大学金陵临床医学院呼吸与危重症学科（展平，吕镗烽，宋勇）; ^3^Department of Respiratory and Critical Care Medicine, Jinling Clinical Medical College, Nanjing University of Chinese Medicine, Nanjing 210002, China

**Keywords:** 恶性胸腔积液, 局部治疗, 进展, 免疫微环境, Malignant pleural effusion, Local therapies, Progress, Immune microenvironment

## Abstract

恶性胸腔积液（malignant pleural effusion, MPE）是指由原发于胸膜或其他部位的恶性肿瘤转移所引起的胸膜腔积液，患者预后差。目前MPE的治疗主要有针对MPE的全身抗肿瘤治疗以及针对原发性肿瘤引起MPE的局部治疗。有关MPE局部治疗的文献报道颇多，方法涉及面广。本文综述近年来针对原发性肿瘤引起的MPE的局部治疗研究进展，总结近年来出现的新药物及新手段。

## Abstract (en)

Malignant pleural effusion (MPE) refers to the accumulation of pleural fluid caused by metastasis from primary pleural malignancies or tumors originating elsewhere. It is associated with a poor prognosis. Current treatment strategies primarily include systemic anti-tumor therapy and local management of MPE based on the primary tumor. Numerous studies have documented diverse approaches for the local control of MPE. This review summarizes recent advances in local treatment strategies for primary tumor-related MPE, highlighting emerging pharmacological agents and innovative techniques.

恶性胸腔积液（malignant pleural effusion, MPE）是指由原发于胸膜或其他部位的恶性肿瘤转移所引起的胸膜腔积液，其确诊依赖胸腔积液样本或胸膜活检组织证实存在恶性肿瘤细胞^[1]^。MPE可见于约20%的恶性肿瘤患者，与多种癌症相关，包括原发性胸膜恶性肿瘤和其他部位肿瘤继发性扩散，包括肺、乳腺和卵巢肿瘤^[2]^。MPE是肿瘤胸膜转移的常见并发症，其形成原因在于肿瘤侵犯导致胸膜毛细血管通透性增高与淋巴回流受阻，致使渗出液在胸膜腔这一潜在密闭空间内异常积聚。大量胸腔积液可压迫同侧肺组织，导致肺不张，并推移纵隔，影响心肺功能，严重影响患者的生存质量和治疗效果^[3]^。MPE患者预后较差，中位生存期通常仅为3-12个月^[4]^。目前，MPE患者的治疗方式包括全身治疗和局部治疗，作为全身治疗的重要补充，MPE患者的局部治疗方式包括胸腔置管引流（indwelling pleural catheter, IPC）、胸膜固定术（pleurodesis）以及胸膜腔内灌注（intrapleural perfusion）等。然而，传统胸膜固定术的成功率并不稳定，其主要影响因素包括术式选择、具体操作技术等。例如，经胸腔镜滑石粉喷洒的成功率较高，可达80%-90%，而经胸腔引流管灌注滑石粉浆的成功率则多在60%-85%^[5,6]^。同时，胸膜物理屏障限制了药物渗透与免疫细胞浸润，胸膜腔内灌注化疗药物因胸膜纤维化及肿瘤异质性难以有效分布。近年研究^[7,8]^发现，免疫微环境在MPE的发生发展过程中发挥关键调控作用，其通过免疫抑制细胞浸润和细胞因子网络介导积液形成、纤维化进程及免疫逃逸，形成恶性循环。随着治疗策略的不断创新，MPE的局部治疗也取得诸多进展。鉴于恶性胸膜间皮瘤所致MPE的治疗与其他病因所致MPE存在较大差异，本文主要围绕常见病因所致MPE的局部治疗进展进行综述。

## 1 MPE的局部治疗

局部治疗作为全身抗肿瘤治疗的有效补充，主要针对原发性肿瘤引起的MPE，旨在缓解患者症状、提高生活质量。常见的局部治疗方法包括IPC、胸膜固定术以及胸膜腔内灌注等。

### 1.1 IPC

IPC通过置入胸腔引流管进行间歇性引流积液，以维持肺的复张。这一治疗适用于有症状的MPE，不依赖于肺是否能够完全复张，尤其是无法耐受或不愿接受胸膜固定术以及既往胸膜固定术治疗失败的患者^[1,9,10]^。Wahidi等^[11]^纳入149例MPE患者，随机分配至每日积极引流组与隔日标准引流组。研究结果显示，每日积极引流组自发性胸膜固定成功率显著高于隔日标准引流组（47% *vs* 24%, OR=2.80, 95%CI: 1.42-5.52, P=0.003）。Muruganandan等^[12]^比较每日积极引流与症状引导下引流MPE的研究，发现两种方案在控制呼吸症状方面没有差异，治疗开始后60天每日积极引流组和症状引导引流组视觉模拟评分（visual analogue scale, VAS）差异几何均数分别为13.1和17.3 mm，几何均数比值为1.32（95%CI: 0.88-1.97, *P*=0.18）。但在治疗开始后60天和6个月后，每日积极引流组的自发性胸膜固定率显著高于症状引导下引流组（治疗开始后60天：37.2% *vs* 11.4%，P=0.0049；治疗开始后6个月后：44.2% *vs* 15.9%，P=0.004），并可以改善患者的生活质量。

OPTIMUM研究^[13]^比较了MPE患者在门诊与住院管理模式下健康相关生活质量（health-related quality of life, HRQoL）和症状的差异，通过对比门诊使用IPC和住院行胸腔引流联合滑石粉胸膜固定术（talc pleurodesis）两种管理策略，评估两者对HRQoL的影响。结果显示，干预后30天，IPC组全球健康状况平均差异为13.11，胸腔引流管联合滑石粉胸膜固定组为10.11，两组患者的健康状况较前均显著改善，组间平均差异为2.06，但无统计学意义（95%CI: -5.86-9.99, P=0.61）。在干预后60和90天的随访中，两组患者的HRQoL均持续保持改善趋势。

这些研究为临床医生制定个体化引流策略提供了有益参考，可根据患者的具体病情做出相应决策。

### 1.2 胸膜固定术

胸膜固定术是指在胸膜的脏层和壁层之间形成联结，使胸膜腔闭合，从而控制MPE的治疗方法。化学性胸膜固定术（chemical pleurodesis）通过注入化学药物，促使胸膜与肺或胸壁之间发生粘连，目的是防止或减少胸腔内积液或肺不张的复发。实施胸膜固定术应满足：胸腔穿刺排液后影像学评估肺可完全复张，或肺大部分可复张（肺萎陷范围<25%）^[10]^。

滑石粉是常见的胸膜硬化剂，多项随机对照研究^[14-16]^评估了IPC与滑石粉胸膜固定术在MPE患者中的疗效和安全性。相较于单独使用IPC，联合滑石粉胸膜固定术能明显提升胸膜固定成功率，尽管该联合方案在呼吸困难缓解、生活质量改善及不良反应方面未展现出显著优势。

为了评估能否在缩短住院时间的同时保持与标准治疗相当的胸膜固定成功率，Psallidas等^[17]^纳入313例患者，比较胸腔超声引导下与标准护理下滑石粉胸腔固定术在MPE患者中的效果，研究结果显示，胸腔超声引导护理和标准护理患者的平均住院时间分别为2和3天（*P*<0.0001），除五维健康量表（EuroQol five-dimension five-level, EQ-5D-5L）评分在3个月时低于标准护理组外，胸腔超声引导护理在全因死亡率、症状评分方面与标准护理无显著差异，然而胸腔超声引导护理显示出明显的成本效益，因此可以考虑在MPE相关胸膜固定术患者中作为标准治疗^[18]^。

化学性胸膜固定术是控制MPE的有效手段，尤其在缓解症状和减少积液复发方面具有明确疗效，已成为国际临床实践中的常用治疗策略。然而，由于滑石粉作为常用硬化剂目前尚未在国内获批用于该适应证，导致这一治疗手段在我国的实际应用受到限制，尚未成为常规临床实践的一部分^[1,10]^。

其他的胸膜硬化剂包括聚维酮碘、博来霉素、多西环素、四环素、自体血液等。自体血液作为一种新型硬化剂，其疗效和安全性与滑石粉相当，30天胸膜固定成功率分别为82%和87%（*P*=0.12），但自体血液作为胸膜硬化剂能够减少发热和疼痛，并缩短住院时间^[19]^。另一项随机对照研究^[20]^比较强力霉素与聚维酮碘通过肋间插管治疗MPE胸膜固定术的疗效和安全性，研究显示两者的胸膜固定成功率相似，分别为96.2%和96.6%（P=0.3）（完全缓解患者：82.7% *vs* 79.3%；部分缓解患者：13.5% *vs* 17.2%）。Paschoalini等^[21]^纳入60例患者，比较硝酸银和滑石粉浆液在MPE胸膜固定术中的疗效，但由于只有少数患者接受硝酸银治疗，且近20%的患者失访，尽管初步研究表明硝酸银具有一定治疗潜力，但因现有研究的样本量有限，且失访率较高，其长期疗效和安全性仍需通过更大规模的临床试验加以验证。

### 1.3 胸膜腔内灌注

胸膜腔内灌注治疗是临床常用的治疗策略，广泛应用于MPE患者，旨在延长患者的生存期，并提高生活质量。目前常用的胸膜腔内灌注治疗主要包括化疗药物、抗血管生成药物、热灌注化疗（hyperthermic intrathoracic chemotherapy, HITHOC）、生物反应调节剂（biological response modifiers, BRM）、免疫检查点抑制剂（immune checkpoint inhibitors, ICIs）、纤维蛋白溶解剂及中药制剂等。

#### 1.3.1 化疗药物

用于胸膜固定的化疗药物能够直接作用于肿瘤细胞，抑制MPE的生成，并可引起胸膜的化学性炎症反应，导致胸膜腔粘连闭合。常见的该类药物包括顺铂、洛铂等铂类制剂。顺铂作为第一代铂类药物，抗肿瘤活性较强，广泛应用于胸膜腔内灌注治疗。然而，由于晚期肺癌患者对化疗并发症的耐受性较差，临床上常采用化疗与其他药物联合灌注，以减毒增效。OK432是从化脓性链球菌（Streptococcus pyogenes）中经青霉素处理制成的免疫调节剂，具有抗肿瘤活性，并可通过促进胸膜纤维化发挥硬化作用^[22]^。一项随机对照研究^[23]^将49例非小细胞肺癌（non-small cell lung cancer, NSCLC）合并MPE的患者随机分为顺铂胸腔内注射组、OK432胸腔内注射组和联合治疗组。三组180天内MPE复发率分别为64.7%、52.9%和13.3%，联合治疗组复发率明显较低（*P*=0.01）。顺铂和OK432胸腔内联合治疗相较于单药顺铂或OK432治疗，在降低MPE复发率上展现出显著优势。另外一项关于洛铂和红霉素胸腔内联合治疗NSCLC合并MPE的临床试验^[24]^，在常规全身化疗的基础上胸腔内注射洛铂和红霉素，分析短期和长期的治疗反应，结果显示，在55例可评估患者中，治疗6周后有效率为81.8%。治疗6和12个月，有效率分别为60.0%和21.8%，1年生存率为83.6%。其不良反应主要表现为短暂、可控的轻中度胸痛与低热，未增加全身化疗毒性，无治疗相关严重并发症或死亡发生。因此，该方案是一种有效且安全的治疗策略。

胸膜腔内灌注化疗在MPE治疗中具有重要地位。以铂类为基础的药物通过直接杀伤肿瘤细胞和促进胸膜粘连的双重机制发挥治疗作用。顺铂联合OK432、洛铂联合红霉素等联合用药方案相较于单药治疗显示出更好的疗效和安全性，能够显著降低MPE复发率，提高患者生活质量。然而，其在改善患者总生存期（overall survival, OS）方面的作用仍不明确，其在临床中的适用性仍需更多高质量研究进一步论证。

#### 1.3.2 抗血管生成药物

血管内皮生长因子（vascular endothelial growth factor, VEGF）在MPE形成过程中发挥关键作用。抗血管生成药物，如重组人血管内皮抑制剂、贝伐珠单抗，通过胸腔内给药，可精准靶向VEGF，阻止其与受体结合，从而抑制血管异常生成，增强血管壁完整性，并有效控制肿瘤进展与胸腔积液的产生^[25,26]^。本团队通过动物实验探讨重组人血管内皮抑制素注射液（恩度）对MPE中血管淋巴管生成的抑制作用。研究采用胸腔注射肺癌细胞株构建小鼠MPE模型，并将动物分为恩度治疗组及多个对照组。通过观察和测量MPE体积及肿瘤灶数量，评估恩度对MPE的抑制效果。实验结果显示，恩度可以显著抑制MPE的形成，减少血管和淋巴管生成，并下调VEGF-A和VEGF-C的表达水平^[27]^。抗血管生成药物与化疗药物联合进行胸腔内灌注给药是肺癌合并MPE的潜在治疗方案。Du等^[28]^纳入72例伴发MPE的晚期转移性NSCLC患者，随机分为贝伐珠单抗联合顺铂组和单药顺铂组，对比两组药物胸腔内灌注控制MPE的效果。联合用药组的总体有效率为83.33%，单药顺铂组则为50.00%（*P*<0.05）。且联合用药组患者的中位无进展生存期（median progression-free survival, mPFS）相较于单药顺铂组显著延长（5.3 *vs* 4.5个月，*P*<0.05）。一项关于铜绿假单胞菌注射液（Pseudomomas aeruginosa injection, PAI）联合恩度治疗MPE的随机试验^[29]^通过将105例患者随机分为PAI联合恩度组、PAI单药组及恩度单药组，比较三组患者的临床疗效和安全性。研究结果显示，PAI联合恩度组的客观缓解率（objective response rate, ORR）和疾病控制率（disease control rate, DCR）分别为68.6%和82.9%，显著高于PAI组的40.0%和57.1%（*P*值分别为0.016、0.039），而恩度组的ORR和DCR为54.3%和65.7%（*P*值分别为0.220、0.179）。此外，获得完全缓解或部分缓解患者中PAI联合恩度组的无复发生存期（relapse-free survival, RFS）为66.1天，显著高于PAI组的49.6天（*P*=0.002）和恩度组的55.7天（*P*=0.044）。PAI联合恩度可改善MPE患者的临床治疗效果，增加患者的RFS。

因此，可在IPC后注入抗血管生成药物联合化疗药物以控制MPE^[1]^。需要注意的是，治疗前应充分评估患者一般状况、凝血功能及药物耐受性，治疗中密切监测不良反应，如出血或胸膜反应等，以实现个体化安全治疗。

#### 1.3.3 HITHOC

HITHOC是一种将治疗肿瘤的热疗和化疗相结合的治疗方式，相较于常温胸腔灌注化疗，HITHOC可提升MPE控制率。

近年来，多项临床研究比较HITHOC与常温胸腹腔灌注化疗对MPE的疗效和安全性。一项多中心随机对照研究^[30]^显示，HITHOC组的ORR达80.70%，显著高于传统化疗组的31.03%（*P*<0.05），但两组的生活质量评分和不良事件发生率方面无显著差异（*P*>0.05）。这表明HITHOC是一种可靠且安全的MPE治疗方式，但其对MPE患者总体生存的影响仍需进一步探究。Li等^[31]^回顾性分析33例初诊伴有MPE的晚期肺癌患者的局部和系统治疗记录，比较HITHOC与IPCD治疗有症状MPE的疗效。术后1个月HITHOC组的MPE控制率为95.7%，与IPCD组的90.0%无明显差异（*P*=0.532）。但3个月时HITHOC组的控制率明显高于IPCD组（69.6% *vs* 30.0%, *P*=0.035）。且亚组分析提示，在接受系统治疗的患者中，联合HITHOC治疗较单纯IPCD可进一步延长患者的胸腔内无进展生存期（intrathoracic PFS, iPFS）（*P*=0.011）和OS（*P*=0.002）。Chen等^[32]^纳入358例终末期恶性肿瘤所致MPE患者，随机分为顺铂联合OK-432胸腔内热疗组和顺铂联合OK-432胸腔内化疗组，探究顺铂联合OK-432胸腔内热疗治疗MPE的安全性和有效性。研究结果显示，顺铂联合OK-432胸腔内热疗组患者的总有效率（93.4%）显著高于顺铂联合OK-432胸腔内化疗组（79.8%）（*P*<0.05），中位生存期分别为8.9和6.2个月（*P*>0.05）。

HITHOC可显著提高ORR，并显示出延长患者PFS的趋势。但其与传统胸腔灌注化疗相比在生存获益方面尚存争议^[33,34]^，作为一种有效的局部治疗选择，其需在具备条件的医疗中心开展。

#### 1.3.4 BRM

BRM是指能够增强、调节或恢复机体免疫应答的一类药物，其成分包括灭活病毒、细菌脂多糖等具有非特异免疫活性的物质。目前应用于胸膜固定术的BRM主要包括A群链球菌制剂（Group A Streptococcus, GAS）、PAI和红色诺卡氏菌细胞壁骨架（Nocardia rubra cell wall skeleton, Nr-CWS）。

一项探究生物大分子Nr-CWS增强自然杀伤（natural killer, NK）细胞介导的癌症免疫治疗的研究^[35]^通过体内和体外模型发现，Nr-CWS可抑制B16F10黑色素瘤模型的肺转移，该效应可能与调控NK细胞功能有关。研究通过组织切片、染色和流式细胞术分析了其对肿瘤生长、转移、细胞凋亡及NK细胞免疫反应的影响。结果表明Nr-CWS通过多途径增强NK细胞活性，包括直接激活NK细胞、刺激细胞因子分泌间接增强其功能以及调节免疫微环境。此外，Nr-CWS还能够迅速消除局部炎症，修复受损组织，并改善免疫微环境，减少炎症因子对NK细胞功能的抑制，从而为NK细胞发挥抗肿瘤作用创造有利条件，为开发新的癌症免疫疗法提供了重要的理论和实验基础。

胸腔内灌注BRM在控制MPE方面显示出一定的潜在疗效，可能通过调节局部免疫微环境、诱导炎症反应或直接杀伤肿瘤细胞等机制发挥作用。然而，目前该类治疗仍缺乏大规模随机对照试验及高质量循证医学证据的支持，其有效性、安全性及适用人群尚未明确。因此，临床医生考虑该治疗方案时，应严格评估患者个体情况，并在多学科讨论基础上酌情使用。

#### 1.3.5 ICIs

ICIs是指一类肿瘤免疫治疗药物，其通过阻断程序性细胞死亡受体1（programmed cell death 1, PD-1）/程序性细胞死亡配体1（programmed cell death ligand 1, PD-L1）抑制剂、细胞毒性T淋巴细胞相关抗原4（cytotoxic T lymphocyte-associated antigen-4, CTLA-4）等免疫检查点蛋白，解除肿瘤对免疫细胞的抑制，从而恢复免疫系统对肿瘤的识别和杀伤能力^[36]^，该类药物的应用，为MPE的治疗提供了新的方向。目前已在临床中使用的包括PD-1/PD-L1抑制剂、CTLA-4抑制剂等。

PD-1抑制剂通过阻断PD-1/PD-L1通路，解除T淋巴细胞的抑制，重新激活免疫系统对肿瘤的攻击。有研究^[37]^通过建立小鼠MPE模型和开展小型临床研究评估胸膜内注射抗PD-1抗体治疗MPE的疗效与安全性。在MPE小鼠模型中，与使用生理盐水的对照组相比，抗PD-1抗体组中位生存期长约1.345倍（39 *vs* 29天，P=0.0098），平均胸腔积液量减少（<300 *vs* >500 μL, *P*=0.003），结节的大小和数量也明显减少（*P*=0.0043），且未观察到明显毒副作用。在9例合并MPE的NSCLC患者中，胸腔内抗PD-1抗体治疗5周后，77.8%的患者胸腔积液量显著减少。给药10周后，胸腔积液控制率为66.7%，且整体安全性良好。在全身抗肿瘤治疗中，与标准多西他赛化疗相比，PD-1抑制剂纳武利尤单抗显著延长了患者的OS和PFS，纳武利尤单抗治疗组和标准化疗组5年合并OS率分别为13.4%和2.6%，PFS率分别为8.0%和0%，且安全性和耐受性更优，生活质量更好^[38]^。但只有少量的小规模探索性临床研究^[39,40]^直接将纳武利尤单抗灌注到胸腔内，且研究结果提示其控制MPE复发的效果不佳。

免疫治疗在MPE的治疗中展现出良好的应用前景，在胸腔灌注治疗方面初步体现出促进免疫激活和局部抗肿瘤效应，但相关临床研究仍较为有限，亟需更多的研究来明确其最佳应用方案。

#### 1.3.6 纤维蛋白溶解剂

胸腔积液中纤维蛋白的沉积以及化疗药物对脏层和壁层胸膜的作用，可能引起化学性炎症，形成分隔性MPE。此时可通过胸腔内注射纤维蛋白溶解剂治疗，常用的药物包括尿激酶、链激酶和组织型纤溶酶原激活物。一项随机对照研究^[41]^探讨链激酶在治疗多房性MPE胸膜固定术中的作用。将40例多房性MPE患者随机分为纤溶组和对照组。比较两组患者24-48 h、48-72 h、24-72 h胸腔引流量、治疗前后胸部计算机断层扫描（computed tomography, CT）图像以及治疗后呼吸困难症状及治疗后复发率。研究结果显示，纤溶组和对照组的平均引流量在24-48 h分别为493和248 mL，48-72 h分别为446和198 mL，24-72 h分别为939和446 mL（*P*<0.001）。两组CT比较，纤溶组有17例（85%）改善超过40%，而对照组仅有7例（35%）改善相同程度（*P*=0.001），两组呼吸困难症状消失率分别为90%和55%（P=0.03），治疗后复发率分别为11%和45%（*P*=0.07），这表明链激酶是一种可靠的治疗选择，且为后续胸膜固定术提供了基础。而另一项尿激酶与安慰剂治疗MPE的随机对照试验^[42]^探讨了胸腔内注射尿激酶对MPE患者呼吸困难和胸膜固定术成功率的影响。将受试者随机分配至胸腔内注射尿激酶或匹配的安慰剂治疗。观察呼吸困难评分、胸膜固定术失败时间等。研究结果显示，胸腔内注射尿激酶在改善患者呼吸困难程度与提高胸膜固定术成功率两方面，均未显示出显著优于安慰剂的疗效。两组间平均呼吸困难评分差异无统计学意义（*P*=0.36），尿激酶组和安慰剂组的胸膜固定术后MPE复发率分别为37%和32%（*P*=0.65），提示胸腔内注射尿激酶不能改善MPE患者的呼吸困难或胸膜固定术后MPE的复发，因此并不建议将其作为胸膜固定术的常规辅助治疗，尽管该疗法在减少胸腔积液和缩短住院时间方面显示出潜在价值，但其确切益处仍需后续研究加以验证。

胸腔内注射纤维蛋白溶解剂治疗MPE有一定的临床支持，但仍需根据患者的具体状况进行个体化治疗，尤其是要考虑到出血风险和感染等潜在副作用。随着更多临床试验的进行，纤维蛋白溶解剂在胸腔积液治疗中的应用可能会得到更广泛的验证和推广。

#### 1.3.7 中药制剂

中医药治疗肺癌伴MPE有多种形式，包括中药汤剂口服、中药外敷、中药制剂胸腔灌注等，对改善症状及减少MPE的产生均有一定的疗效^[43]^。一项关于芒硝大黄外敷联合胸腔内化疗治疗MPE的前瞻性随机对照临床试验^[44]^纳入84例患者，将其分为芒硝大黄外敷联合胸腔内化疗治疗组和单纯胸腔内化疗对照组，对胸腔引流液放置后的临床结果进行评价，研究结果显示治疗组的总有效率显著高于对照组（66.67% *vs* 54.76%, *P*<0.05），且临床症状改善较大，未见不良反应的发生。作为西医治疗的辅助手段，中药外敷通过渗透压作用促使组织内水分外渗吸收及促进局部循环减少积液、改善症状，是治疗MPE的有效方法，但目前相关研究仍以小样本探索为主，其临床疗效的可靠性尚需通过大样本随机对照试验进一步验证。

## 2 新型局部治疗技术

### 2.1 新型生物工程药物

MPE的进展与肿瘤微环境的免疫抑制特性及全身给药后药物在胸膜病灶部位的低递送效率密切相关。传统的局部治疗手段，如胸膜腔穿刺术与胸膜固定术，虽可缓解症状，但在诱导抗肿瘤免疫应答及改善患者OS方面存在局限。近年来，基于生物工程策略的新型药物与免疫疗法协同应用，通过逆转肿瘤微环境免疫抑制状态并特异性激活抗肿瘤免疫构建出一种新的治疗策略^[8,45]^。

MPE作为一种典型的“冷肿瘤”，具有多种免疫抑制细胞富集，并含有大量免疫抑制细胞因子。因此，寻求一种能有效地将“冷肿瘤”变为“热肿瘤”的新型胸腔内治疗方法，对于改善MPE的免疫治疗至关重要^[46]^。Song等^[47]^一项关于通过使用转化纳米治疗的方法来重新调节肺腺癌MPE中的免疫微环境以提高免疫治疗效果的研究，使用硒纳米颗粒作为转化纳米治疗的载体，将硒纳米通过靶向递送系统引导到肺腺癌患者的胸腔积液中。实验结果表明，硒纳米颗粒能够显著改善肺腺癌患者胸腔积液中的免疫微环境，增加免疫细胞的活性和浸润，促进肿瘤抗原的呈递和识别，并增强免疫细胞对肿瘤细胞的杀伤作用。此外，它还能够减少免疫抑制细胞的数量和功能，从而增强免疫治疗的效果。另一项关于胸膜内纳米免疫治疗促进先天和适应性免疫反应以增强抗PD-L1治疗MPE的研究^[48]^，开发了一种载环二核苷酸（liposomal nanoparticle loaded with cyclic dinucleotide, LNP-CDN）的脂质体纳米颗粒，用于靶向激活巨噬细胞和树突状细胞（dendritic cells, DCs）中干扰素基因信号的刺激因子，建立小鼠MPE模型，在胸膜内给药时，它们会诱导MPE的转录环境发生剧烈变化，减轻积液和胸膜肿瘤中的免疫冷型MPE，并联合免疫治疗与PD-L1抑制剂治疗有效减小MPE体积并抑制胸膜腔和肺实质中的肿瘤生长，从而显著延长了携带MPE小鼠的生存期。此外，在临床MPE样本中观察到的LNP-CDN免疫学效应，证实了其作为胸膜腔内免疫疗法的潜在价值，为肺癌合并MPE的治疗提供了新的思路。

作为天然药物传递系统，细胞膜来源的微颗粒（microparticles, MPs）是细胞间通讯的关键介质。有研究^[49-51]^探索肿瘤细胞源性MPs（tumor cell-derived MPs, TMPs）在MPE中的治疗潜力。在小鼠Lewis肺癌和结肠腺癌细胞诱导的MPE模型中，包装化疗药物甲氨蝶呤（methotrexate, MTX）的TMPs明显限制MPE的产生，并展现了潜在生存益处。基于TMPs-MTX的潜在益处和最小毒性，研究者进行了一项人体胸腔内给药单剂量自体TMPs包装MTX（autologous tumor cell-derived MPs packaging methotrexate, ATMPs-MTX）的研究^[51]^，通过对11例晚期肺癌伴MPE患者的研究结果分析发现，注射ATMPs-MTX具有可行性和安全性，靶向恶性细胞和免疫抑制微环境的ATMPs可能是治疗恶性肿瘤的一个有希望的方式。

基于细菌的胸腔内治疗通过免疫刺激或细胞毒作用发挥了抗MPE的作用。有研究团队^[52]^设计了一种乳酸菌益生菌（probiotic lactococcus lactis, FOLactis），表达FMS样酪氨酸激酶3（FMS-like tyrosine kinase 3）和共刺激因子OX40配体（co-stimulator OX40 ligands）的融合蛋白。FOLactis可激活肿瘤抗原特异性免疫反应，并通过瘤内传递显示全身抗肿瘤效果。研究团队发现，胸膜腔内注射FOLactis（intrapleural administration of FOLactis, I-Pl FOLactis）不仅能明显抑制MPE和胸膜肿瘤结节，而且能显著延长携带MPE小鼠的存活时间。与野生型细菌组相比，FOLactis组肿瘤引流淋巴结中CD103^+^ DCs的比例增加了3倍。增强的DCs募集促进效应记忆T细胞和CD8^+^ T细胞的渗透，以及NK细胞的激活和巨噬细胞对M1的极化。且与PD-1抑制剂联用进一步增强了I-Pl FOLactis的抗肿瘤效果。这种基于FOLactis的创新胸腔内治疗策略，显示出显著的疗效和良好的生物安全性。

### 2.2 加压胸腔喷雾化疗（pressurized intrathoracic aerosol chemotherapy, PITAC）

PITAC是一种微创、局部的靶向化疗技术，旨在直接向胸膜腔内部喷洒高浓度的化疗药物，以杀死肿瘤细胞、控制积液并实现胸膜固定^[53]^。一项评估PITAC治疗MPE安全性和可行性的I期临床试验^[54]^对20例患者至少进行2次PITAC治疗，该研究预计PITAC对患者及医护人员均具有良好的安全性和临床可行性，并在减少MPE方面展现出积极效果。另一项针对NSCLC胸膜转移（pleural carcinomatosis from NSCLC, PC-NSCLC）的PITAC的试点研究^[55]^，评估PITAC诱导胸膜固定、减少MPE复发的疗效，并借助患者原代细胞培养分析其局部抗肿瘤效果。该研究中，7例患者均成功接受PITAC治疗，术中无并发症发生，术后30天死亡率为0%，未出现全身性毒性反应或化疗药物相关超敏反应。所有患者在治疗后30天内均实现有效胸膜固定，且在3和6个月随访中MPE未复发。细胞实验进一步表明PITAC处理后癌细胞生长也受到显著抑制。PITAC为MPE的治疗提供了一种创新性的解决方案，但由于其尚未普及，长期疗效和最佳用药方案仍需更多临床数据支持。

### 2.3 新型泵系统

一种新颖的允许液体从胸膜空间移动到膀胱的泵系统，可能对复发性MPE的管理有作用。研究团队^[56]^设计了一种名为Pleurapump的泵系统，可以将液体从胸腔腔隙转移到膀胱，并通过正常排尿排出体外。研究首先进行了动物实验，成功将Pleurapump植入4只猪体内，并通过多次流体输送循环测试了泵系统的功能。在动物实验中，研究团队观察到泵系统在不同呼吸条件下均能成功运作并实现液体输送。随后，进行了第一阶段的人体研究，共招募了2例患者，在胸腔镜检查过程中，将Pleurapump植入患者的腹部右侧皮下组织中，并通过胸腔导管和膀胱导管将液体引流到泵系统中。手术后，患者可以通过无线方式检索植入泵的运行信息，并根据需要进行治疗调整。通过这项研究，研究团队成功验证了Pleurapump泵系统在动物模型和人体中的可行性和安全性，为MPE的管理提供了一种新的选择，也有望改善患者的症状和生活质量。

## 3 小结与展望

近年来，MPE的局部治疗策略不断演进，已不再局限于传统的姑息性引流，而是逐步形成注重治疗效率与患者生活质量的多元化、个体化模式。胸腔内灌注治疗，尤其是局部免疫治疗的应用，展现出显著潜力，其通过调节胸腔免疫微环境，增强抗肿瘤免疫应答，从而实现对MPE的有效控制。此外，癌症疫苗、基于生物材料的药物递送系统等前沿技术也为MPE治疗提供了新的可能性。未来，有必要继续推进针对MPE的临床研究，为MPE这一晚期肿瘤常见并发症提供更加有效、精准的治疗选择。
